# Human cytomegalovirus infection and coronary heart disease: a systematic review

**DOI:** 10.1186/s12985-018-0937-3

**Published:** 2018-02-06

**Authors:** Yu Du, Guangxue Zhang, Zhijun Liu

**Affiliations:** 1 Department of Microbiology, Weifang Medical University, Weifang, 261053 China; 2 Department of Clinical Laboratory, Shandong Qingzhou Rongjun Hospital, Qingzhou, 262500 China

**Keywords:** Human cytomegalovirus, Coronary heart disease, Infection

## Abstract

**Background:**

Human cytomegalovirus (HCMV) infection is closely associated with coronary heart disease.

**Main body of the abstract:**

In 1987, Adam et al. were the first to report an association between HCMV infection and atherosclerosis (AS), and later, many serum epidemiology and molecular biology studies showed that HCMV-infected endothelial cells play an important role in the development of AS. As patients with HCMV are generally susceptible to coronary heart disease, and with the increasing elderly population, a review of recent studies focusing on the relationships of HCMV infection and coronary heart disease is timely and necessary.

**Short conclusion:**

The role of HCMV infection in the development of AS needs further study, since many remaining issues need to be explored and resolved. For example, whether HCMV promotes the development of coronary AS, and what the independent factors that lead to coronary artery AS by viral infection are. A comprehensive understanding of HCMV infection is needed in order to develop better strategies for preventing AS.

## Background

Coronary atherosclerotic heart disease, also known as coronary heart disease (CHD), involves the coronary and other circulations [1–3]. Atherosclerosis (AS) causes vascular stenosis and/or obstruction, resulting in myocardial ischemia [4, 5] and hypoxia [6] and ultimately CHD with loss to society [7, 8] and even death [9]. AS is prevalent in the elderly: in Western society it is the primary cause of death, and in China, the incidence is ~ 80% in those over the age of 60 years [10–12]. It is showing an increasing trend along with population aging.

The World Health Organization divides CHD into 5 types [3]: silent myocardial ischemia (occult CHD), angina pectoris, myocardial infarction, ischemic heart failure (ischemic heart disease), and sudden death. Clinically, it is divided into stable CHD and acute coronary syndrome. Although epidemiological studies have shown that classical risk factors such as high blood lipids [13–15], high blood pressure [16, 17], smoking [18–23], obesity [24–29], and diabetes [30–33] lead to CHD, its etiology is not fully understood. In recent years, these traditional risk factors have been effectively reduced, but the incidence of AS is still high [33–35]. And 30–50% of patients with AS lack these classic risk factors, indicating that other unknown factors are involved in its pathogenesis.

Further studies have revealed that infection is closely associated with CHD. Epidemiological studies have shown that pathogens such as *Chlamydia* [36–42], *Cytomegalovirus* (CMV) [43–46], and *Helicobacter pylori* are involved in the occurrence and development of AS [47–51]. CMV infection has received increased attention since Frabricant successfully established an experimental AS model in chickens infected with CMV [52]. In recent years, many serological and molecular-biological studies have shown that human CMV (HCMV) infection of endothelial cells (ECs) plays an important role in the development of AS [53–59]. Several pathological and animal models have suggested that HCMV infection is involved in the pathogenesis of CHD. Here, we review research progress in the relationship between HCMV infection and CHD.

## Correlation between cytomegalovirus and coronary heart disease

In the 1940s, Paterson and Cottral noted a very close relationship between herpes virus infection and AS, and in 1983, Melniek et al. investigated possible viruses associated with human AS lesions but failed. Later, they removed plaques and surrounding tissues, cultured them, and used immunological techniques to detect HCMV antigen. They found that 52% of AS lesions were HCMV antigen-positive while non-AS tissues were only 29% positive, suggesting that HCMV is an initiating factor for AS [60]. The results of Horvath et al. suggested that HCMV and Epstein-Barr virus occur in the arterial wall, which is thus a potential site of persistence of these viruses. They also found a significant association between the presence of HCMV DNA in the aortic wall and AS [61].

Along with further research, related studies on serological epidemiology, molecular biology, and animal experiments have also indicated that HCMV infection is closely associated with the progression of AS.

### Sero-epidemiological evidence

In 1987, Adam first reported that CMV infection is associated with AS: the HCMV infection rate and the antibody titer were significantly higher in an AS group than in a non-AS group [62]. Meanwhile, the antibody level did not vary with the cholesterol level, suggesting that HCMV infection is involved in the occurrence of AS and CHD. Eryol investigated HCMV-IgG in 179 patients suspected of having CHD [63], and after coronary angiography, 123 were diagnosed with the disease. Only 6 individuals were IgG-negative and 87 had a high titer (> 8 μg/ml). After excluding CHD caused by the traditional risk factors, a high antibody titer remained a significant factor. A study of 14,153 cases showed that patients who underwent cardiopulmonary resuscitation were positive for HCMV antibody. These patients had high levels of C-reactive protein (CRP) and showed 30.1% higher mortality and 29.5% higher cardiovascular mortality than those who were HCMV-negative and had low CRP levels [64].

In addition, CMV is associated with ischemic heart disease (IHD) among organ transplant recipients. Gkrania-Klotsas et al. showed that CMV IgG antibody levels are associated with incident IHD compared to seronegativity in the population-based EPIC-Norfolk study [65]. The prospective AS risk in communities (ARIC) study of CMV, herpes simplex virus 1, and CHD showed that high levels of CMV antibodies are significantly associated with incident CHD. Infection with CMV, particularly in susceptible disease states such as diabetes, may be an important risk factor for CHD [66] and there is at least a modest association between CMV and asymptomatic carotid wall thickening, consistent with early AS [67].

### Molecular biological evidence

Direct evidence for a link between HCMV and AS has come from recent molecular biology studies. The polymerase chain reaction (PCR) technique has been used to detect the HCMV immediate early (IE) and late (L) genes. Xenaki et al. detected HCMV in the peripheral blood of 40 patients with CHD using PCR [68]. The results showed that the positivity rate of HCMV in the CHD group was significantly higher than that in the control group. Chen detected HCMV IE genes and L gene fragments using PCR and in situ hybridization, and found that the positive rate in the AS group was significantly higher than that in the non-AS group [58]. In addition, the viral DNA was mainly concentrated in the nucleus and the endothelial layer as well as in lesions of the smooth muscle layer, suggesting a possible pathogenic effect of HCMV infection in AS. Reinhardt detected HCMV in HCMV-infected cultured human renal arterial cells and, using immunohistochemistry and in situ hybridization, found gradual HCMV infiltration, indicating that the activation state of virus infection causes inflammation of the artery [69]. In addition, HCMV infection increases the incidence of coronary AS in patients after cardiac transplantation, suggesting that HCMV infection is closely associated with AS [70–73].

### Animal models of atherosclerosis caused by CMV

Fabricant showed that chickens infected​ with Marek Disease Virus (MDV) had AS lesions mainly involving arteries of the heart, abdominal cavity, stomach, and mesenteries; in contrast, no lesions were found in uninfected chickens [74]. Early-stage MDV antigen was concentrated at the sites of vascular injury, while in the advanced stage the antigen was present in the smooth muscle cells (SMCs) at the periphery of the plaque, consistent with the hypothesis that HCMV leads to AS. The formation of plaques in vascular SMCs (VSMCs), characterized by excessive proliferation and increased fat, was similar to the histological characteristics in human AS. In 1993, Bruggeman et al. confirmed that soon after heart transplantation in rats (after 1 day), rat CMV infection resulted in adventitial inflammatory cell infiltration, hyperplasia of intimal SMCs, and the formation of endometrial AS lesions [75]. This demonstrated that CMV is a pathogenic factor for AS. In 1994, they revealed that 9-(1,3-dihydroxy-2-propoxymethyl)guanine inhibits AS alterations primarily by reducing the infectious virus and thereby the inflammatory response in the allograft vascular wall; another possibility is a direct antiproliferative effect on SMC replication [76]. Also, the preventive application of ganciclovir sodium reduced the rat CMV infection that induced the formation of AS after cardiac transplantation [77]. They detected CMV using real-time PCR in the vascular ECs of rabbits infected with CMV for 3, 5, 14, and 35 days [78]. These results showed that CMV infection mainly affects the proliferation of VSMCs, leading to intimal thickening. Besides, Alber et al. reported an accelerated occurrence of AS in an apolipoprotein-E-deficient model of herpesvirus-infected mice and that antiviral therapy significantly reduced the incidence of AS [79].

### Different views

Although most studies have suggested that HCMV infection is associated with the risk of CHD, many epidemiological studies have shown opposite results. In 900 patients with coronary AS, Adler reported that the IgG-positive rate and IgG titer were not correlated with AS [80]. In a 12-year prospective study, Ridker discussed the relationship between HCMV and the future risk of myocardial infarction and stroke. After correcting for the traditional cardiovascular risk factors, no evidence for a relationship was found [81]. In a controlled study of HCMV infection in patients with CHD in Germany, 312 40–60 year-old patients (coronary artery stenosis > 50%) and 479 healthy volunteers were included. After correcting for covariates, there was no statistically significant difference in HCMV-IgG antibody, only for CRP [82]. Also, no hemorheology or inflammatory markers related to CMV serology were found.

## Possible mechanism of HCMV infection developing to atherosclerosis

Previous studies have suggested that viral infection is a cause of AS. The biological characteristics of HCMV are consistent with the pathogenesis of AS; systemic HCMV infection leads to sub-clinical inflammation; and HCMV infects ECs, leading to cellular injury and metabolic changes [83]. Viruses from ECs then infect SMCs, and the latent or persistent infection in SMCs leads to proliferation and the accumulation of cholesterol and cholesterol esters. In cases of low immunity, latent infection is repeatedly activated, resulting in repeated damage to the arterial wall. In infected cells, abnormal apoptotic changes may play an important role in the pathogenesis. HCMV infection induces apoptosis in cultured ECs [84]. The apoptosis rate increases throughout the course of infection. In the early stage of infection, the rate is low, while the rate increases later to enhance proliferation. Finally, in the last stage of infection, the apoptosis rate decreases, which favors latent viruses.

### Cytomegalovirus infection leads to the inflammatory reaction and immune responses

HCMV infection is systemic, and can cause subclinical inflammation, which is one of the important factors in AS. CRP reflects the degree of inflammatory activity and is also a sensitive index of tissue damage [85–87]. The study by De Backer et al. showed that patients with CHD had significantly higher CRP than the negative control group [88]. In addition, the HCMV-IgM antibody titer was positively correlated with hsCRP. CRP regulates the expression of many AS factors, and shows opsonization similar to IgG and complement. CRP promotes the activation of local complement, leading to tissue damage; the serum CRP levels are increased in stable angina pectoris, unstable angina pectoris, and acute myocardial infarction, suggesting that they reflect severe injury in unstable angina pectoris plaques [89]. A prospective study confirmed that hsCRP is an independent predictor of myocardial infarction and continuing high CRP indicates an increased risk of coronary events [90]. Biasucci et al. investigated coronary ischemic events in a follow-up of patients with stable angina pectoris of CHD over a year, and found that the CRP concentrations were < 2.6, 2.6–8.6, and > 8.6 mg/L in the blood of patients with incidence rates of coronary ischemic events of 13%, 42%, and 67%, respectively (normal values < 3 mg/L) [91].

HCMV infection can cause a chronic immune inflammatory reaction [92]. The latent HCMV infection is periodically reactivated, resulting in a chronic immune response or inflammatory response that is damaging to the vascular endothelium and inner membrane, resulting in SMC proliferation and mutation. The formation of antibody immune complexes of HCMV antigen deposited in the vascular wall in AS lesions can induce vascular ECs, macrophages, foam cells, SMCs, and T lymphocytes to express the monocyte proteins CCL-2, − 3, − 4, and − 5 and macrophage colony-stimulating factor [61, 93]. Moreover, they also stimulate macrophages to produce and release of interleukins IL-1, − 6, − 8, − 10, and − 12, tumor necrosis factor alpha (TNF-α), and other inflammatory cell factors, which cause cellular and humoral immune responses, and accelerate the release of CRP to induce an inflammatory chain-reaction. The activation of blood mononuclear cells induces migration into the intima. Among these, CCL-2 is the most important and the most potent inducer of monocyte migration [94, 95]. These cells in vitro stimulate the expression of CCL-2 via oxidized low-density lipoprotein (OX-LDL) [96]. Another study showed that, due to periodic HCMV activation in arterial SMCs caused by local immune responses and inflammation, SMCs show degeneration and apoptosis caused by inflammatory substances, leading to instability in plaques, which are prone to rupture and bleeding, leading to acute coronary syndrome [97].

Another important factor is TNF-α. After necrosis of ECs, TNF-α is significantly increased, causing acute vascular inflammation. Jeong et al. investigated the relationship between anti-HCMV antibody and carotid artery AS in South Korea [46]. A higher level of TNF-α and a greater intima-media thickness is associated with AS development. And TNF-α as an inflammatory factor, causing acute vascular inflammation, is important in the transition from AS and stable CHD to acute coronary syndrome. Thus, TNF-α reflects the level of inflammation in chronic AS infection. In addition, TNF-α promotes the expression of intercellular adhesion molecule-1 (ICAM-1), which assists the binding of ECs to leukocytes. TNF-α also promotes the early progression of AS by hardening blood vessels through its enhancement of collagen deposition by fibroblasts.

In 1996, Speir et al. showed that SMCs produce reactive oxygen species (ROS), including peroxide and hydroxyl free-radicals, leading to the activation of nuclear factor κB, and the intracellular ROS stimulates the expression and replication of HCMV genes to strengthen viral functions [98]. Under physiological conditions, free radicals are eliminated in vivo via antioxidant mechanisms, but under oxidative stress, an imbalance of ROS occurs. ROS increases in the vascular wall and the excess ROS modifies the endothelium to generate OX-LDL, which increases the permeability of vascular ECs, leukocyte infiltration, and cell proliferation, as well as changing cell death and apoptosis, interfering with intracellular signal transduction, and mediating the migration, differentiation, and secretion of cytokines by mononuclear cells. Studies have shown that HCMV products also activate the cyclooxygenase-2 (COX-2) promoter, increasing arachidonic acid release [99, 100]. ROS are produced via a COX-2-dependent pathway and cause vascular endothelial injury, platelet activation, platelet adhesion and aggregation, enhanced release, and increased tissue-type plasminogen activator activity, resulting in AS plaque thrombosis.

### Endothelial injury

In vivo studies have revealed that HCMV infection of the vessel wall affects various cells including monocytes/macrophages, SMCs, and ECs [93]. Vascular ECs function as a barrier, as well as receiving and transmitting information, and secreting factors to maintain vascular integrity, regulate vascular permeability, and maintain blood flow. Moreover, they synthesize prostacyclin, thromboxane A2, endothelin, and their surface thrombomodulin. The adenosine diphosphate enzyme plays a role in platelet aggregation, thrombosis, and cell adhesion. ECs provide an antithrombotic surface and prevent the activation of coagulation factors and platelets [101]. EC damage is considered to be the initial step in AS formation. Endothelial dysfunction causes a change of endothelial permeability, promotes the proliferation of VSMCs, affects lipid metabolism, and influences cytokines and other inflammatory substances to induce the expression of leukocyte adhesion molecules and their products. It also induces inflammation of vascular endothelia, and affects blood coagulation and the fibrinolytic balance [99].

The replication of HCMV in vascular ECs is the key point of persistent viral infection, transmission, and disease onset, and is the most direct cause of endothelial dysfunction and apoptosis. Ryckman et al. reported that three protein complexes of the HCMV UL128, UL130, and UL131 genes encode the viral envelope glycoprotein gH/gL that promotes the transport of HCMV into vascular ECs [102]. Their findings showed that UL128, UL130, and UL131 must all bind simultaneously onto gH/gL for the production of complexes that can function in entry into epithelial cells and ECs. Viral infection inevitably leads to abnormal metabolism and function in vascular ECs. These pathologies are due in important part to the ability of HCMV to enter and replicate in diverse cell types including ECs, SMCs, fibroblasts, and monocytes/macrophages, which may increase AS in the general population. Gharavi et al. showed that CMV stimulates the host to produce antibodies, mainly via combination with the receptors on the surface of ECs [103]. Specific antibodies bind the corresponding cell membrane antigen and active complement, leading directly to cellular damage or enhancing monocyte and neutrophil chemotaxis to induce EC injury via immune regulation, immune adhesion, and antibody-dependent cell-mediated cytotoxicity.

Bason et al. found that the IgG antibody to virus infection develops a cross-reaction with serum heat-shock protein, leading to metabolic changes in uninfected ECs [57]. The expression of vascular endothelial growth factor and adhesion molecules in HCMV-negative patients is significantly lower than in positive patients, and their expression is proportional to CRP. The enhancement of expression can promote the adhesion of ECs, monocytes, neutrophils, and lymphocytes and cause inflammation to damage ECs, which enhances the entry of monocytes into subcutaneous tissue. The infected EC releases virus particles to increase the local virus titer which becomes a further invasive source for VSMCs and persistent viremia. An influence on the metabolism and function of SMCs may be caused by direct interactions among cells and the release of soluble cytokines, such as IL-6, IL-8, basic fibroblast growth factor [104], cig5/viperin [56], and platelet-derived growth factor (PDGF) [105].

In HCMV infection of ECs, miRNA-US25–1 expression is up-regulated. MiR-US25–1 regulates viral and host cell replication cycles, and its upregulation reduces EC viability without causing apoptosis and long-term inflammation of the vessel wall. The studies of Fan and others have shown that miR-US25–1 transfected into vascular ECs, the first combination of 5’-UTR binding site, so that the expression of HEK293 decreased [20]. These proteins are mainly cyclin-dependent kinase and the anti-apoptotic protein BRCC. The expression of the BRCC gene decreases, which could affect the repair of DNA damage and decrease the mRNA and protein content of the host cells, as well as damaging the ECs infected by HCMV, becoming a basis for AS [106].

### CMV infection affects lipid metabolism and causes lipid deposition

After CMV infection, cholesterol and triglycerides increase significantly in the host, and accumulate in the arterial wall without an effect of diet. Hepatic lipase (HL), lipoprotein lipase, and lecithin-cholesterol acyltransferase are considered to be the key enzymes in lipid metabolism; changes in these enzymes lead directly to disorders of lipid metabolism. HCMV infection modifies the host lipidome profile and the expression of several genes and microRNAs involved in cholesterol metabolism. In mice, murine CMV infection elevates plasma triglycerides but does not affect the level and functionality of high-density lipoprotein. HCMV, through its protein US28, reorganizes lipid rafts and disturbs cellular cholesterol metabolism [107]. In addition, CMV infection induces the pro-inflammatory cytokine IL-6, which then triggers ECs to release CCL-2, which recruits more monocytes and T-cells into the vessel wall thereby exacerbating local inflammation, and thus atherogenesis [108]. IL-6 and other pro-inflammatory molecules influence the cholesterol and lipid metabolism of ECs. In vivo experiments have shown that high levels of IL-6 stimulate trophoblast fatty-acid accumulation, which contributes to an excessive nutrient transfer in conditions associated with elevated IL-6 [109]. The synthesis of intracellular cholesterol results mainly from a series of enzymatic reactions involving hydroxymethylglutaryl coenzyme A (HMG-CoA) synthetase and HMG-CoA reductase. Thus, HCMV can infect VSMCs and the infection affects gene expression associated with cellular lipid metabolism, and this is involved in the occurrence and development of AS [110].

Studies have shown that after infection with HCMV, the HMG-CoA synthase, HMG-CoA reductase, and acetyl-CoA acetyltransferase genes are expressed in infected VSMCs, confirming the upregulation of genes associated with the synthesis of cholesterol and cholesteryl ester expression. A study by Xie showed that VSMCs infected with HCMV have significantly increased expression of LDL receptor-associated proteins 10, 11, and 12, and intracellular expression of the HMG-CoA synthase and HMG-CoA reductase genes associated with cholesterol synthesis are also up-regulated. Meanwhile, the apolipoprotein A1 and apolipoprotein M genes are downregulated, and these are closely associated with anti-AS activity [110]. During inflammation and infection, by activating the positive acute response gene HMG-CoA reductase (encoding the rate-limiting enzyme of cholesterol synthesis) or by acting on cellular factors that reduce the cholesterol metabolism of 7a–cholesterol hydroxylase (CYP7A1), gene transcription increases the serum triglyceride and cholesterol levels [111]. After HCMV infection of Hep cells, activity of the CYP7A1 promoter and the activity of G2 are significantly decreased, and the infection interferes with cholesterol metabolism by affecting the activity of the hepatic CYP7A1 promoter. Simultaneously, HCMV infection also effects the intracellular lipid transporter [112]. The ATP-binding cassette ABCA1 is a lipid transporter that is very important for HDL production, and the liver is rich in ABCA1 expression, indicating that it plays a major role in HDL balance. Sanchez and Dong demonstrated decreased protein expression of ABCA1 in the early stage of HCMV infection. There is an inverse relationship between levels of serum HDL and cardiovascular disease and because ABCA1 functions in the initial stages of HDL synthesis, this protein is considered to be athero-protective. These findings further confirm that HCMV infection negatively regulates the ABCA1 carrier [113].

A recent study showed that upregulation of single-stranded DNA-binding protein-1 (SSBP1) inhibits the expression of AS-associated low-density lipoprotein receptor, scavenger receptor class B, 3-hydroxy-3-methylglutaryl coenzyme A reductase, and cholesteryl ester transfer protein, along with the accumulation of lipids in cells. The inhibition of SSBP1 by HCMV infection promotes this lipid accumulation [114], which depends on the uptake of OX-LDL, a process in which the scavenger receptor (SR) has been postulated to play an important role. Zhou et al. demonstrated that HCMV infection of human SMCs increases modified LDL uptake and stimulates the expression of class A SR gene mRNA [115].

### Promotion of the proliferation and migration of VSMCs

HCMV can lead to developmental and metabolic disorders of VSMCs, in which changes in proliferation and metabolism are not only the basic components of AS, but also the first components of the disease. HCMV has the characteristics of a tumor DNA virus [116–118]. The latent virus can be activated and recur periodically under conditions such as hypertension and hyperlipidemia so as to promote the proliferation of SMCs. The proliferation of SMCs is very important in the process of AS plaque formation [119]. The main cellular component of the fibrous plaque is SMCs, which not only synthesize connective tissue matrix but also phagocytize lipids in the endothelial formation of foamy cells. With the gradual increase, swelling, and disintegration of foamy cells, AS plaques remain unstable.

Tang-Feldman et al. [28] showed that HCMV infects human cells after the release of arachidonic acid, catalyzes the production of ROS by COX, and that IE-72/IE-86 activate the promoter of COX-2, increasing ROS production in infected cells. These two pathways stimulate the production of ROS to activate the inactive state of NF-κB in the cytoplasm, which elicits immune and inflammation-related expression of multiple cytokines such as IL-6 and IL-8 [120]. Prochnau et al. showed that a series of inflammatory factors such as IL-6, IL-8, and PDGF-AA are significantly up-regulated in SMCs after 24 and 72 h of HCMV infection. These cytokines induce oxygen free radicals, damage the vascular endothelium, activate platelets, enhance platelet adhesion, release, and aggregation, and enhance the activity of histone prothrombin activator, leading to thrombosis at the AS plaque. IL-6 induces ECs to release CCL-2, recruiting more monocytes and lymphocytes to the intima, exacerbating the local inflammatory response. IL-8 activates chemotactic neutrophils, and activated neutrophil oxidative bursts produce large amounts of ROS, stimulating more NF-κB and inflammatory factor production. The various factors constitute a complex network of mutual induction and mutual regulation, and jointly promote the development of AS [104].

P53 is one of the most important tumor-suppressor genes associated with human tumors. The expression of p53 is important in cell-cycle regulation, blocking the cell cycle in G1, and inducing apoptosis. SMCs grown from lesions express the HCMV protein IE-86 and large amounts of p53. HCMV infection of cultured SMCs enhances p53 accumulation, which is temporally correlated with IE-86 expression [121]. Both of the HCMV genes IE-72 and IE-86 are sufficient to alter the cell cycle and generate an environment conducive to proliferation [122]. Plus, p53 is also responsible for the anti-apoptotic properties of HCMV-infected cells [123]. The IE-86 protein encoded by HCMV combines with intracellular p53 protein and inhibits its function, so it cannot play its role in apoptosis regulation, leading to hyperproliferation and vascular angiostenosis [124]. Moreover, IE-2 up-regulates the expression of the anti-apoptotic genes *mcl-1* and *bcl-2*, reducing the number of apoptotic cells and not only aggravating inflammation, but also causing the accumulation of susceptible SMCs, leading to the occurrence of vascular stenosis, an important mechanism in AS. HCMV infection also changes cellular metabolism, promotes lipid deposition, and upregulates the SR expression in VSMCs, promoting the development of vascular injury [125]. Reinhardt showed that human VSMCs infected with HCMV have enhanced expression of PDGF receptors [126]. This increases the uptake of OX-LDL into VSMCs and the expression of SRs promotes the formation of AS plaques. Meanwhile, PDGF also inhibits collagenase by upregulating the expression of tissue inhibitor of metalloproteinase and reducing the degradation of extracellular matrix.

### HCMV promotes coagulation and increases thrombosis

HCMV infection leads to increased CRP, which induces the expression of tissue factor in monocytes, initiates the coagulation process, and increases the risk of thrombosis; elevated CRP also enhances the coronary risk [127]. CRP is a chemotactic factor for fibrinogen, resulting in the accumulation of reactive T lymphocytes, increased platelet activity, and imbalance of the coagulation and fibrinolysis system, increasing the incidence of arterial thrombosis and acute coronary syndrome [128]. Vascular ECs produce and release nitric oxide (NO), an endothelial factor, to inhibit platelet aggregation and the adhesion of monocytes and macrophages [129]. HCMV infection leads to reduced NO synthesis and secretion, switching the platelet status from anticoagulant to pro-coagulant, thus increasing both vascular endothelial adhesion and thrombosis [54]. The activation of latent HCMV infection can periodically affect vascular ECs and cause high expression of platelet alpha-granule membrane protein-140, E-selectin, intercellular adhesion molecule-1 (ICAM-1), vascular cell adhesion molecules, and other tissue factors, which greatly change EC adhesion and resistance to anticoagulation and increase the binding of ICAM-1 among white blood cells, platelets, and ECs. Moreover, the platelets change from the anticoagulant to the pro-coagulant state, so platelet adhesion and aggregation occur. This is a key step in early inflammatory vascular injury. HCMV US2 is a multifunctional degradation hub which modulates diverse immune pathways involved in antigen presentation, natural killer cell activation, migration, and coagulation. This highlights the impact of US2 on HCMV pathogenesis [130]. Finally, HCMV-infected ECs also promote the formation of coagulation factors and tissue factors. In addition, monocytes play a central role in the process of viral dissemination, whereas ECs may serve as a viral reservoir, maintaining persistent infection in HCMV-infected AS patients following the primary infection. Persistent infection leads to dysfunction of ECs and activates pro-inflammatory signaling involving NFκB, specificity protein 1, and phosphatidylinositol 3-kinase, as well as expression of the platelet-derived growth factor receptor [93].

Studies have revealed roles of monocytes in viral dissemination and crosstalk between these cells and ECs during HCMV infection. Studies have shown that both OX-LDL/ lectin-like OX-LDL receptor 1 and CCL-2 attract monocytes to ECs in an initial step in atherogenesis [131, 132]. Similarly, Bolovan-Fritts and Spector reported a major pathogenic effect in which EC damage and loss follow the induction of fractalkine and the up-regulation of cell adhesion markers in the presence of peripheral blood mononuclear cells (PBMCs) from donors with a high CMV-specific T-cell frequency [133]. So, when CMV infects ECs, PBMCs can be productively infected by contact with CMV-infected ECs, as shown by co-culturing PBMCs with CMV-infected endothelial monolayers [134]. Gerna’s study also demonstrated a role of monocytes to viral dissemination. In primary infection, both epithelial cells and ECs can be infected first. Then virus transport occurs via leukocytes such as monocytes and polymorphonuclear leukocytes. As monocytes differentiate to macrophages, they become highly susceptible to human CMV replication inside organ tissues, while polymorphonuclear leukocytes are active in viral-capture from infected endothelial vascular cells and transporting them to distant sites [135]. Interestingly, Guetta et al. found that freshly-isolated human monocytes infected with endothelium-passaged CMV can transmit CMV to co-cultured ECs or SMCs [136], which enriches the hypothesis of monocyte-EC interactions and crosstalk in the progress of viral infection.

## Conclusions

Over the past 30 years, there has been extensive discussion of whether pathogens are involved in AS pathology. Studies provide powerful evidence regarding the involvement of HCMV in the formation of CHD and AS. The AS-associated factors, from molecular biological evidence and animal models, have revealed the relationship between AS and HCMV infection. The development of AS is closely related to inflammatory reactions and the immune response, endothelial injury, lipid deposition, metabolic disorders of VSMCs, and coagulation thrombosis.

Recent studies have shown that HCMV-induced vascular AS begins with an inflammatory response to HCMV infection, particularly in immunodeficient patients. HCMV infection can affect nonspecific immunity and adaptive immune control in healthy hosts, but it has the characteristics of long-term persistent latency, threatening and acting on the host at all times. In cases of low immunity, HCMV activation causes a series of inflammatory responses. In addition, transplant patients are particularly susceptible to HCMV infection-related diseases. It is clear that future research should focus on the underlying diseases caused by the indirect effects of the virus, and HCMV-related AS is one such disease. Although considerable experimental and epidemiological evidence suggests that HCMV is associated with AS, the molecular mechanisms linking the two are unclear. It has been suggested that HCMV infection is involved in the molecular biological mechanism of AS formation, and this will be helpful for developing new drugs to prevent or delay AS formation.

## Abbreviations

ABCA1: ATP-binding cassette
ARIC: Atherosclerosis risk in communities
AS: Atherosclerosis
CCL-2: Chemokine ligand 2
CHD: Coronary heart disease
CMV: Cytomegalovirus
COX-2: Cyclooxygenase-2
CRP: C-reactive protein
CYP7A1: Cholesterol metabolism of 7a–cholesterol hydroxylase
EC: Endothelial cells
HCMV: Human cytomegalovirus
HL: Hepatic lipase
HMG-CoA: Hydroxymethylglutaryl coenzyme A
ICAM-1: Intercellular adhesion molecule-1
IE: Immediate early
IE: Immediate early response protein
IHD: Ischemic heart disease
L: Late
MDV: Marek disease virus
NO: Nitric oxide
OX-LDL: Oxidized low-density lipoprotein
PCR: Polymerase chain reaction
PDGF: Platelet-derived growth factor
PDGF: Platelet-derived growth factor
ROS: Reactive oxygen species
SR: Scavenger receptor
SSBP1: Single-stranded DNA-binding protein-1
TNF: Tumor necrosis factor
VSMCs: Vascular smooth muscle cells

## References

[1] Tyagi M, Fteropoulli T, Hurt CS, Hirani SP, Rixon L, Davies A, Picaut N, Kennedy F, Deanfield J, Cullen S, Newman SP (2017). Cognitive dysfunction in adult CHD with different structural complexity. Cardiol Young.

[2] Arbab-Zadeh A, Fuster V (2016). The risk continuum of atherosclerosis and its implications for defining CHD by coronary angiography. J Am Coll Cardiol.

[3] Wong ND (2014). Epidemiological studies of CHD and the evolution of preventive cardiology. Nat Rev Cardiol.

[4] Takada S, Kurita Y, Imanishi T, Otsuka A, Shinbo H, Furuse H, Nakanishi T, Suzuki A, Takase H, Ozono S (2011). Arteriosclerosis related factors had no clinical significant correlation with resistive index in symptomatic benign prostatic hyperplasia. Urology.

[5] Ramalli EL, Braga LH, Evora PM, Albuquerque AA, Celotto AC, Mota AL, Evora PR (2011). Absence of arteriosclerosis in intramyocardial coronary arteries: a mystery to be solved?. Rev Bras Cir Cardiovasc.

[6] Ma L, Zhang J, Liu Y (2016). Roles and mechanisms of obstructive sleep Apnea-Hypopnea syndrome and chronic intermittent hypoxia in atherosclerosis: evidence and prospective. Oxidative Med Cell Longev.

[7] Ohnuki T, Takahashi W, Ohnuki Y, Kawada S, Takizawa S (2013). Significance of the presence of metabolic syndrome in patients with asymptomatic arteriosclerosis affecting the aorta and the cerebral, extra-cranial carotid and coronary arteries. Intern Med.

[8] Japan Arteriosclerosis Longitudinal Study G (2008). Japan arteriosclerosis longitudinal study-existing cohorts combine (JALS-ECC): rationale, design, and population characteristics. Circ J.

[9] Fishbein MC, Fishbein GA (2015). Arteriosclerosis: facts and fancy. Cardiovasc Pathol.

[10] Liu Y, Qi LT, Ma W, Yang Y, Meng L, Zhang BW, Huo Y (2013). Relationship between radial augmentation index and other indices for evaluating arteriosclerosis. Beijing Da Xue Xue Bao.

[11] Ren S, Qian S, Wang W, Liu J, Liu P (2013). Prospective study of sarpogrelate hydrochloride on patients with arteriosclerosis obliterans. Ann Thorac Cardiovasc Surg.

[12] Bonner G, Gysan DB, Sauer G (2005). Prevention of arteriosclerosis. Importance of the treatment of arterial hypertension. Z Kardiol.

[13] Shimizu R, Torii H, Yasuda D, Hiraoka Y, Furukawa Y, Yoshimoto A, Iwakura T, Matsuoka N, Tomii K, Kohara N (2016). Comparison of serum lipid management between elderly and non-elderly patients with and without coronary heart disease (CHD). Prev Med Rep.

[14] Ahola TL, Kantola IM, Puukka P, Kattainen A, Klaukka T, Reunanen A, Jula AM (2010). Positive change in the utilization of antihypertensive and lipid-lowering drugs among adult CHD patients in Finland: results from a large national database between 2000 and 2006. Eur J Cardiovasc Prev Rehabil.

[15] Koro CE, L'Italien GJ, Fedder DO (2004). Major CHD risk factors predominate among African-American women who are eligible for lipid-lowering drug therapy under the new ATP III guidelines. Eur J Cardiovasc Prev Rehabil.

[16] Eisenmann JC, Wrede J, Heelan KA (2005). Associations between adiposity, family history of CHD and blood pressure in 3-8 year-old children. J Hum Hypertens.

[17] Brandon LJ, Mullis RM, Jonnalagadda SS, Hughes MH (2005). Relationships and CHD risks of BMI, lipoproteins, lipids, and blood pressure in African-American men and women. Prev Med.

[18] Asia Pacific Cohort Studies C (2009). Impact of cigarette smoking on the relationship between body mass index and coronary heart disease: a pooled analysis of 3264 stroke and 2706 CHD events in 378579 individuals in the Asia Pacific region. BMC Public Health.

[19] Lightwood JM, Coxson PG, Bibbins-Domingo K, Williams LW, Goldman L (2009). Coronary heart disease attributable to passive smoking: CHD policy model. Am J Prev Med.

[20] Frothingham SM, Smith PO, Payne TJ, Meadows SE (2008). Clinical inquiries: how much does smoking cessation cut CHD risk?. J Fam Pract.

[21] Fowler G (1997). Passive smoking was associated with an increased risk of CHD. Evid Based Cardiovasc Med.

[22] Wienke A, Herskind AM, Christensen K, Skytthe A, Yashin AI (2005). The heritability of CHD mortality in danish twins after controlling for smoking and BMI. Twin Res Hum Genet.

[23] Diverse Populations C (2004). Smoking, body weight, and CHD mortality in diverse populations. Prev Med.

[24] Kang MY, Hong YC (2016). Inter-correlation between working hours, sleep duration, obesity, and 10-year risk for CHD. Am J Ind Med.

[25] Lin GM, Li YH, Lai CP, Lin CL, Wang JH (2015). The obesity-mortality paradox in elderly patients with angiographic coronary artery disease: a report from the ET-CHD registry. Acta Cardiol.

[26] Kwagyan J, Retta TM, Ketete M, Bettencourt CN, Maqbool AR, Xu S, Randall OS (2015). Obesity and cardiovascular diseases in a high-risk population: evidence-based approach to CHD risk reduction. Ethn Dis.

[27] Logue J (2011). Obesity is an independent risk factor for death from CHD. Practitioner.

[28] Ghosh A, Bose K, Das Chaudhuri AB (2003). Association of food patterns, central obesity measures and metabolic risk factors for coronary heart disease (CHD) in middle aged Bengalee Hindu men, Calcutta, India. Asia Pac J Clin Nutr.

[29] Brotons C, Soler-Soler J (2000). Obesity, Cinderella of CHD risk factors. Eur Heart J.

[30] Gao N, Yuan Z, Tang X, Zhou X, Zhao M, Liu L, Ji J, Xue F, Ning G, Zhao J (2016). Prevalence of CHD-related metabolic comorbidity of diabetes mellitus in northern Chinese adults: the REACTION study. J Diabetes Complicat.

[31] Lockyer M (2014). Women with diabetes at greater risk of CHD than men. Practitioner.

[32] Soedamah-Muthu SS, Masset G, Verberne L, Geleijnse JM, Brunner EJ (2013). Consumption of dairy products and associations with incident diabetes, CHD and mortality in the Whitehall II study. Br J Nutr.

[33] Southerland JH, Moss K, Taylor GW, Beck JD, Pankow J, Gangula PR, Offenbacher S (2012). Periodontitis and diabetes associations with measures of atherosclerosis and CHD. Atherosclerosis.

[34] Kucharska-Newton A, Griswold M, Yao ZH, Foraker R, Rose K, Rosamond W, Wagenknecht L, Koton S, Pompeii L, Windham BG. Cardiovascular disease and patterns of change in functional status over 15 years: findings from the atherosclerosis risk in communities (ARIC) study. J Am Heart Assoc. 2017;6:e004144. 10.1161/JAHA.116.004144. 10.1161/JAHA.116.004144 PMC5523991 28249844

[35] Weber C, Shantsila E, Hristov M, Caligiuri G, Guzik T, Heine GH, Hoefer IE, Monaco C, Peter K, Rainger E (2016). Role and analysis of monocyte subsets in cardiovascular disease. Joint consensus document of the European Society of Cardiology (ESC) working groups “Atherosclerosis & Vascular Biology” and “thrombosis”. Thromb Haemost.

[36] Yazouli LE, Criscuolo A, Hejaji H, Bouaaza M, Elmdaghri N, Alami AA, Amraoui A, Dakka N, Radouani F. Molecular characterization of Chlamydia pneumoniae associated to atherosclerosis. Pathog Dis. 2017;75. 10.1093/femspd/ftx039. 10.1093/femspd/ftx039 28387800

[37] Patil K, Campbell LA, Rosenfeld ME, Paik J, Brabb T, O'Brien KD, Maggio-Price L, Hsu CC (2016). Effects of Murine Norovirus on Chlamydia pneumoniae-accelerated atherosclerosis in ApoE(−/−) mice. Comp Med.

[38] Filardo S, Di Pietro M, Farcomeni A, Schiavoni G, Sessa R (2015). Chlamydia pneumoniae-mediated inflammation in atherosclerosis: a meta-analysis. Mediat Inflamm.

[39] Zafiratos MT, Manam S, Henderson KK, Ramsey KH, Murthy AK. CD8+ T cells mediate Chlamydia pneumoniae-induced atherosclerosis in mice. Pathog Dis. 2015;73. 10.1093/femspd/ftv052. 10.1093/femspd/ftv052 PMC4830221 26220574

[40] Sorrentino R, Yilmaz A, Schubert K, Crother TR, Pinto A, Shimada K, Arditi M, Chen S (2015). A single infection with Chlamydia pneumoniae is sufficient to exacerbate atherosclerosis in ApoE deficient mice. Cell Immunol.

[41] Kreutmayer S, Csordas A, Kern J, Maass V, Almanzar G, Offterdinger M, Ollinger R, Maass M, Wick G (2013). Chlamydia pneumoniae infection acts as an endothelial stressor with the potential to initiate the earliest heat shock protein 60-dependent inflammatory stage of atherosclerosis. Cell Stress Chaperones.

[42] Zhao X, Bu DX, Hayfron K, Pinkerton KE, Bevins CL, Lichtman A, Wiedeman J (2012). A combination of secondhand cigarette smoke and Chlamydia pneumoniae accelerates atherosclerosis. Atherosclerosis.

[43] Wang Z, Cai J, Zhang M, Wang X, Chi H, Feng H, Yang X (2016). Positive expression of human cytomegalovirus Phosphoprotein 65 in atherosclerosis. Biomed Res Int.

[44] Zhang J, Liu YY, Sun HL, Li S, Xiong HR, Yang ZQ, Xiang GD, Jiang XJ (2015). High human cytomegalovirus IgG level is associated with increased incidence of diabetic atherosclerosis in type 2 diabetes mellitus patients. Med Sci Monit.

[45] Heybar H, Alavi SM, Farashahi Nejad M, Latifi M (2015). Cytomegalovirus infection and atherosclerosis in candidate of coronary artery bypass graft. Jundishapur J Microbiol.

[46] Jeong SJ, Ku NS, Han SH, Choi JY, Kim CO, Song YG, Kim JM (2015). Anti-cytomegalovirus antibody levels are associated with carotid atherosclerosis and inflammatory cytokine production in elderly Koreans. Clin Chim Acta.

[47] Xu Y, Wang Q, Liu Y, Cui R, Lu K, Zhao Y (2016). Association between helicobacter pylori infection and carotid atherosclerosis in patients with vascular dementia. J Neurol Sci.

[48] Blum A (2015). Helicobacter pylori and atherosclerosis. Isr Med Assoc J.

[49] Karbasi-Afshar R, Khedmat H, Izadi M (2015). Helicobacter pylori infection and atherosclerosis: a systematic review. Acta Med Iran.

[50] He C, Yang Z, Lu NH (2014). Helicobacter pylori-an infectious risk factor for atherosclerosis?. J Atheroscler Thromb.

[51] Kang SG, Chung WC, Song SW, Joo KR, Lee H, Kang D, Lee JS, Lee KM (2013). Risk of atherosclerosis and helicobacter pylori infection according to CD14 Promotor polymorphism in healthy Korean population. Gastroenterol Res Pract.

[52] Fabricant CG, Fabricant J, Minick CR, Litrenta MM (1983). Herpesvirus-induced atherosclerosis in chickens. Fed Proc.

[53] Sacre K, Hunt PW, Hsue PY, Maidji E, Martin JN, Deeks SG, Autran B, McCune JM (2012). A role for cytomegalovirus-specific CD4+CX3CR1+ T cells and cytomegalovirus-induced T-cell immunopathology in HIV-associated atherosclerosis. AIDS.

[54] Lunardi C, Dolcino M, Peterlana D, Bason C, Navone R, Tamassia N, Tinazzi E, Beri R, Corrocher R, Puccetti A (2007). Endothelial cells’ activation and apoptosis induced by a subset of antibodies against human cytomegalovirus: relevance to the pathogenesis of atherosclerosis. PLoS One.

[55] Grahame-Clarke C (2005). Human cytomegalovirus, endothelial function and atherosclerosis. Herpesviridae.

[56] Olofsson PS, Jatta K, Wagsater D, Gredmark S, Hedin U, Paulsson-Berne G, Soderberg-Naucler C, Hansson GK, Sirsjo A (2005). The antiviral cytomegalovirus inducible gene 5/viperin is expressed in atherosclerosis and regulated by proinflammatory agents. Arterioscler Thromb Vasc Biol.

[57] Bason C, Corrocher R, Lunardi C, Puccetti P, Olivieri O, Girelli D, Navone R, Beri R, Millo E, Margonato A (2003). Interaction of antibodies against cytomegalovirus with heat-shock protein 60 in pathogenesis of atherosclerosis. Lancet.

[58] Chen R, Xiong S, Yang Y, Fu W, Wang Y, Ge J (2003). The relationship between human cytomegalovirus infection and atherosclerosis development. Mol Cell Biochem.

[59] Melnick JL, Adam E, DeBakey ME (1996). Cytomegalovirus and atherosclerosis. Arch Immunol Ther Exp.

[60] Melnick JL, Petrie BL, Dreesman GR, Burek J, McCollum CH, DeBakey ME (1983). Cytomegalovirus antigen within human arterial smooth muscle cells. Lancet.

[61] Horvath R, Cerny J, Benedik J, Hokl J, Jelinkova I, Benedik J (2000). The possible role of human cytomegalovirus (HCMV) in the origin of atherosclerosis. J Clin Virol.

[62] Adam E, Melnick JL, Probtsfield JL, Petrie BL, Burek J, Bailey KR, McCollum CH, DeBakey ME (1987). High levels of cytomegalovirus antibody in patients requiring vascular surgery for atherosclerosis. Lancet.

[63] Eryol NK, Kilic H, Gul A, Ozdogru I, Inanc T, Dogan A, Topsakal R, Basar E (2005). Are the high levels of cytomegalovirus antibodies a determinant in the development of coronary artery disease?. Int Heart J.

[64] Simanek AM, Dowd JB, Pawelec G, Melzer D, Dutta A, Aiello AE (2011). Seropositivity to cytomegalovirus, inflammation, all-cause and cardiovascular disease-related mortality in the United States. PLoS One.

[65] Gkrania-Klotsas E, Langenberg C, Sharp SJ, Luben R, Khaw KT, Wareham NJ (2012). Higher immunoglobulin G antibody levels against cytomegalovirus are associated with incident ischemic heart disease in the population-based EPIC-Norfolk cohort. J Infect Dis.

[66] Sorlie PD, Nieto FJ, Adam E, Folsom AR, Shahar E, Massing M (2000). A prospective study of cytomegalovirus, herpes simplex virus 1, and coronary heart disease: the atherosclerosis risk in communities (ARIC) study. Arch Intern Med.

[67] Sorlie PD, Adam E, Melnick SL, Folsom A, Skelton T, Chambless LE, Barnes R, Melnick JL (1994). Cytomegalovirus/herpesvirus and carotid atherosclerosis: the ARIC study. J Med Virol.

[68] Xenaki E, Hassoulas J, Apostolakis S, Sourvinos G, Spandidos DA (2009). Detection of cytomegalovirus in atherosclerotic plaques and nonatherosclerotic arteries. Angiology.

[69] Reinhardt B, Vaida B, Voisard R, Keller L, Breul J, Metzger H, Herter T, Baur R, Luske A, Mertens T (2003). Human cytomegalovirus infection in human renal arteries in vitro. J Virol Methods.

[70] Streblow DN, Kreklywich CN, Andoh T, Moses AV, Dumortier J, Smith PP, Defilippis V, Fruh K, Nelson JA, Orloff SL (2008). The role of angiogenic and wound repair factors during CMV-accelerated transplant vascular sclerosis in rat cardiac transplants. Am J Transplant.

[71] De La Melena VT, Kreklywich CN, Streblow DN, Yin Q, Cook JW, Soderberg-Naucler C, Bruggeman CA, Nelson JA, Orloff SL (2001). Kinetics and development of CMV-accelerated transplant vascular sclerosis in rat cardiac allografts is linked to early increase in chemokine expression and presence of virus. Transplant Proc.

[72] Sambiase NV, Higuchi ML, Nuovo G, Gutierrez PS, Fiorelli AI, Uip DE, Ramires JA (2000). CMV and transplant-related coronary atherosclerosis: an immunohistochemical, in situ hybridization, and polymerase chain reaction in situ study. Mod Pathol.

[73] Pouria S, State OI, Wong W, Hendry BM (1998). CMV infection is associated with transplant renal artery stenosis. QJM.

[74] Fabricant CG, Fabricant J, Litrenta MM, Minick CR (1978). Virus-induced atherosclerosis. J Exp Med.

[75] Lemstrom KB, Bruning JH, Bruggeman CA, Lautenschlager IT, Hayry PJ (1993). Cytomegalovirus infection enhances smooth muscle cell proliferation and intimal thickening of rat aortic allografts. J Clin Invest.

[76] Lemstrom KB, Bruning JH, Bruggeman CA, Koskinen PK, Aho PT, Yilmaz S, Lautenschlager IT, Hayry PJ (1994). Cytomegalovirus infection-enhanced allograft arteriosclerosis is prevented by DHPG prophylaxis in the rat. Circulation.

[77] Lemstrom KB, Koskinen PK, Bruning JH, Bruggeman CA, Lautenschlager IT, Hayry PJ (1994). Effect of ganciclovir prophylaxis on cytomegalovirus-enhanced allograft arteriosclerosis. Transpl Int.

[78] Kloppenburg G, de Graaf R, Herngreen S, Grauls G, Bruggeman C, Stassen F (2005). Cytomegalovirus aggravates intimal hyperplasia in rats by stimulating smooth muscle cell proliferation. Microbes Infect.

[79] Alber DG, Powell KL, Vallance P, Goodwin DA, Grahame-Clarke C (2000). Herpesvirus infection accelerates atherosclerosis in the apolipoprotein E-deficient mouse. Circulation.

[80] Adler SP, Hur JK, Wang JB, Vetrovec GW (1998). Prior infection with cytomegalovirus is not a major risk factor for angiographically demonstrated coronary artery atherosclerosis. J Infect Dis.

[81] Ridker PM, Hennekens CH, Stampfer MJ, Wang F (1998). Prospective study of herpes simplex virus, cytomegalovirus, and the risk of future myocardial infarction and stroke. Circulation.

[82] Rothenbacher D, Hoffmeister A, Bode G, Wanner P, Koenig W, Brenner H (1999). Cytomegalovirus infection and coronary heart disease: results of a german case-control study. J Infect Dis.

[83] Rahbar A, Soderberg-Naucler C (2005). Human cytomegalovirus infection of endothelial cells triggers platelet adhesion and aggregation. J Virol.

[84] Taveira A, Ponroy N, Mueller NJ, Millard AL (2014). Entry of human cytomegalovirus into porcine endothelial cells depends on both the cellular vascular origin and the viral strain. Xenotransplantation.

[85] Niyi-Odumosu FA, Bello OA, Biliaminu SA, Owoyele BV, Abu TO, Dominic OL (2017). Resting serum concentration of high-sensitivity C-reactive protein (hs-CRP) in sportsmen and untrained male adults. Niger J Physiol Sci.

[86] Mukherjee S, Barman S, Mandal NC, Bhattacharya S (2014). Anti-bacterial activity of Achatina CRP and its mechanism of action. Indian J Exp Biol.

[87] Varol E, Aksoy F, Icli A, Arslan A, Yuksel O, Ersoy IH, Varol S, Dogan A (2012). Increased plasma neopterin and hs-CRP levels in patients with endemic fluorosis. Bull Environ Contam Toxicol.

[88] De Backer J, Mak R, De Bacquer D, Van Renterghem L, Verbraekel E, Kornitzer M, De Backer G (2002). Parameters of inflammation and infection in a community based case-control study of coronary heart disease. Atherosclerosis.

[89] Alavi SM, Adel SM, Rajabzadeh AR (2011). An evidence against the effect of chronic cytomegalovirus infection in unstable angina pectoris. Acta Med Iran.

[90] Ridker PM (2007). C-reactive protein and the prediction of cardiovascular events among those at intermediate risk: moving an inflammatory hypothesis toward consensus. J Am Coll Cardiol.

[91] Biasucci LM, Liuzzo G, Grillo RL, Caligiuri G, Rebuzzi AG, Buffon A, Summaria F, Ginnetti F, Fadda G, Maseri A (1999). Elevated levels of C-reactive protein at discharge in patients with unstable angina predict recurrent instability. Circulation.

[92] van de Berg PJ, Heutinck KM, Raabe R, Minnee RC, Young SL, van Donselaar-van der Pant KA, Bemelman FJ, van Lier RA, ten Berge IJ (2010). Human cytomegalovirus induces systemic immune activation characterized by a type 1 cytokine signature. J Infect Dis.

[93] Popovic M, Smiljanic K, Dobutovic B, Syrovets T, Simmet T, Isenovic ER (2012). Human cytomegalovirus infection and atherothrombosis. J Thromb Thrombolysis.

[94] Muller WA (2001). New mechanisms and pathways for monocyte recruitment. J Exp Med.

[95] Bakst RL, Xiong H, Chen CH, Deborde S, Lyubchik A, Zhou Y, He S, McNamara WF, Lee SY, Olson OC (2017). Inflammatory monocytes promote perineural invasion via CCL2-mediated recruitment and cathepsin B expression. Cancer Res.

[96] Silva AR, Pacheco P, Vieira-de-Abreu A, Maya-Monteiro CM, D'Alegria B, Magalhaes KG, de Assis EF, Bandeira-Melo C, Castro-Faria-Neto HC, Bozza PT (2009). Lipid bodies in oxidized LDL-induced foam cells are leukotriene-synthesizing organelles: a MCP-1/CCL2 regulated phenomenon. Biochim Biophys Acta.

[97] Izadi M, Fazel M, Saadat SH, Nasseri MH, Ghasemi M, Dabiri H, Aryan RS, Esfahani AA, Ahmadi A, Kazemi-Saleh D (2012). Cytomegalovirus localization in atherosclerotic plaques is associated with acute coronary syndromes: report of 105 patients. Methodist Debakey Cardiovasc J.

[98] Speir E, Shibutani T, Yu ZX, Ferrans V, Epstein SE (1996). Role of reactive oxygen intermediates in cytomegalovirus gene expression and in the response of human smooth muscle cells to viral infection. Circ Res.

[99] Schroer J, Shenk T (2008). Inhibition of cyclooxygenase activity blocks cell-to-cell spread of human cytomegalovirus. Proc Natl Acad Sci U S A.

[100] Zhu H, Cong JP, Yu D, Bresnahan WA, Shenk TE (2002). Inhibition of cyclooxygenase 2 blocks human cytomegalovirus replication. Proc Natl Acad Sci U S A.

[101] Davignon J, Ganz P (2004). Role of endothelial dysfunction in atherosclerosis. Circulation.

[102] Ryckman BJ, Rainish BL, Chase MC, Borton JA, Nelson JA, Jarvis MA, Johnson DC (2008). Characterization of the human cytomegalovirus gH/gL/UL128-131 complex that mediates entry into epithelial and endothelial cells. J Virol.

[103] Gharavi AE, Pierangeli SS, Harris EN (2003). Viral origin of antiphospholipid antibodies: endothelial cell activation and thrombus enhancement by CMV peptide-induced APL antibodies. Immunobiology.

[104] Prochnau D, Straube E, Figulla HR, Rodel J (2012). Supra-additive expression of interleukin-6, interleukin-8 and basic fibroblast growth factor in vascular smooth muscle cells following coinfection with Chlamydia pneumoniae and cytomegalovirus as a novel link between infection and atherosclerosis. Can J Infect Dis Med Microbiol.

[105] Koskinen PK, Kallio EA, Bruggeman CA, Lemstrom KB (1997). Cytomegalovirus infection enhances experimental obliterative bronchiolitis in rat tracheal allografts. Am J Respir Crit Care Med.

[106] Grey F, Tirabassi R, Meyers H, Wu G, McWeeney S, Hook L, Nelson JA (2010). A viral microRNA down-regulates multiple cell cycle genes through mRNA 5'UTRs. PLoS Pathog.

[107] Low H, Mukhamedova N, Cui HL, McSharry BP, Avdic S, Hoang A, Ditiatkovski M, Liu Y, Fu Y, Meikle PJ (2016). Cytomegalovirus restructures lipid rafts via a US28/CDC42-mediated pathway, enhancing cholesterol efflux from host cells. Cell Rep.

[108] Rott D, Zhu J, Zhou YF, Burnett MS, Zalles-Ganley A, Epstein SE (2003). IL-6 is produced by splenocytes derived from CMV-infected mice in response to CMV antigens, and induces MCP-1 production by endothelial cells: a new mechanistic paradigm for infection-induced atherogenesis. Atherosclerosis.

[109] Lager S, Jansson N, Olsson AL, Wennergren M, Jansson T, Powell TL (2011). Effect of IL-6 and TNF-alpha on fatty acid uptake in cultured human primary trophoblast cells. Placenta.

[110] Li L, Li Y, Dai Z, Liu M, Wang B, Liu S, Wang L, Chen L, Tan Y, Wu G (2016). Lipid metabolism in vascular smooth muscle cells Infuenced by HCMV infection. Cell Physiol Biochem.

[111] Dobesh PP, Swahn SM, Peterson EJ, Olsen KM (2010). Statins in sepsis. J Pharm Pract.

[112] Khovidhunkit W, Kim MS, Memon RA, Shigenaga JK, Moser AH, Feingold KR, Grunfeld C (2004). Effects of infection and inflammation on lipid and lipoprotein metabolism: mechanisms and consequences to the host. J Lipid Res.

[113] Sanchez V, Dong JJ (2010). Alteration of lipid metabolism in cells infected with human cytomegalovirus. Virology.

[114] Guo N, Zhang N, Yan L, Cao X, Lv F, Wang J, Wang Y, Cong H (2017). Down-regulation of single-stranded DNA-binding protein 1 expression induced by HCMV infection promotes lipid accumulation in cells. Braz J Med Biol Res.

[115] Zhou YF, Guetta E, Yu ZX, Finkel T, Epstein SE (1996). Human cytomegalovirus increases modified low density lipoprotein uptake and scavenger receptor mRNA expression in vascular smooth muscle cells. J Clin Invest.

[116] Pasquereau S, Al Moussawi F, Karam W, Diab Assaf M, Kumar A, Herbein G (2017). Cytomegalovirus, macrophages and breast cancer. Open Virol J.

[117] Joseph GP, McDermott R, Baryshnikova MA, Cobbs CS, Ulasov IV (2017). Cytomegalovirus as an oncomodulatory agent in the progression of glioma. Cancer Lett.

[118] Schlick K, Grundbichler M, Auberger J, Kern JM, Hell M, Hohla F, Hopfinger G, Greil R (2015). Cytomegalovirus reactivation and its clinical impact in patients with solid tumors. Infect Agent Cancer.

[119] Li Y, Guo Y, Chen Y, Wang Y, You Y, Yang Q, Weng X, Li Q, Zhu X, Zhou B (2015). Establishment of an interleukin-1beta-induced inflammation-activated endothelial cell-smooth muscle cell-mononuclear cell co-culture model and evaluation of the anti-inflammatory effects of tanshinone IIA on atherosclerosis. Mol Med Rep.

[120] Tang-Feldman YJ, Lochhead SR, Lochhead GR, Yu C, George M, Villablanca AC, Pomeroy C (2013). Murine cytomegalovirus (MCMV) infection upregulates P38 MAP kinase in aortas of Apo E KO mice: a molecular mechanism for MCMV-induced acceleration of atherosclerosis. J Cardiovasc Transl Res.

[121] Speir E, Modali R, Huang ES, Leon MB, Shawl F, Finkel T, Epstein SE (1994). Potential role of human cytomegalovirus and p53 interaction in coronary restenosis. Science.

[122] Castillo JP, Yurochko AD, Kowalik TF (2000). Role of human cytomegalovirus immediate-early proteins in cell growth control. J Virol.

[123] Tanaka K, Zou JP, Takeda K, Ferrans VJ, Sandford GR, Johnson TM, Finkel T, Epstein SE (1999). Effects of human cytomegalovirus immediate-early proteins on p53-mediated apoptosis in coronary artery smooth muscle cells. Circulation.

[124] Poole E, Bain M, Teague L, Takei Y, Laskey R, Sinclair J (2012). The cellular protein MCM3AP is required for inhibition of cellular DNA synthesis by the IE86 protein of human cytomegalovirus. PLoS One.

[125] Zhou YF, Yu ZX, Wanishsawad C, Shou M, Epstein SE (1999). The immediate early gene products of human cytomegalovirus increase vascular smooth muscle cell migration, proliferation, and expression of PDGF beta-receptor. Biochem Biophys Res Commun.

[126] Reinhardt B, Mertens T, Mayr-Beyrle U, Frank H, Luske A, Schierling K, Waltenberger J (2005). HCMV infection of human vascular smooth muscle cells leads to enhanced expression of functionally intact PDGF beta-receptor. Cardiovasc Res.

[127] Kervinen H, Palosuo T, Manninen V, Tenkanen L, Vaarala O, Manttari M (2001). Joint effects of C-reactive protein and other risk factors on acute coronary events. Am Heart J.

[128] Corrado E, Novo S (2005). Role of inflammation and infection in vascular disease. Acta Chir Belg.

[129] Sadowitz B, Maier KG, Gahtan V (2010). Basic science review: Statin therapy--part I: the pleiotropic effects of statins in cardiovascular disease. Vasc Endovasc Surg.

[130] Hsu JL, van den Boomen DJ, Tomasec P, Weekes MP, Antrobus R, Stanton RJ, Ruckova E, Sugrue D, Wilkie GS, Davison AJ (2015). Plasma membrane profiling defines an expanded class of cell surface proteins selectively targeted for degradation by HCMV US2 in cooperation with UL141. PLoS Pathog.

[131] Froberg MK, Dannen D, Adams A, Parker-Thornburg J, Kolattukudy P (2006). Murine cytomegalovirus infection markedly reduces serum MCP-1 levels in MCP-1 transgenic mice. Ann Clin Lab Sci.

[132] Chen K, Chen J, Liu Y, Xie J, Li D, Sawamura T, Hermonat PL, Mehta JL (2005). Adhesion molecule expression in fibroblasts: alteration in fibroblast biology after transfection with LOX-1 plasmids. Hypertension.

[133] Bolovan-Fritts CA, Spector SA (2008). Endothelial damage from cytomegalovirus-specific host immune response can be prevented by targeted disruption of fractalkine-CX3CR1 interaction. Blood.

[134] Waldman WJ, Knight DA, Huang EH, Sedmak DD (1995). Bidirectional transmission of infectious cytomegalovirus between monocytes and vascular endothelial cells: an in vitro model. J Infect Dis.

[135] Gerna G (2012). Endothelial cells and CMV dissemination. Future Microbiol.

[136] Guetta E, Guetta V, Shibutani T, Epstein SE (1997). Monocytes harboring cytomegalovirus: interactions with endothelial cells, smooth muscle cells, and oxidized low-density lipoprotein. Possible mechanisms for activating virus delivered by monocytes to sites of vascular injury. Circ Res.

